# Engineered CRISPR/Cas13d Sensing hTERT Selectively Inhibits the Progression of Bladder Cancer *In Vitro*

**DOI:** 10.3389/fmolb.2021.646412

**Published:** 2021-03-19

**Authors:** Chengle Zhuang, Changshui Zhuang, Qun Zhou, Xueting Huang, Yaoting Gui, Yongqing Lai, Shangqi Yang

**Affiliations:** ^1^ Department of Urology, Peking University Shenzhen Hospital, Shenzhen, China; ^2^ Department of Urology, Union Shenzhen Hospital, Huazhong University of Science and Technology, Shenzhen, China; ^3^ Department of Urology, the Affiliated Nanhua Hospital of University of South China, Hengyang, China; ^4^ Department of Nephrorheumatology, Shenzhen Yantian District People’s Hospital, Shenzhen, China

**Keywords:** CRISPR/Cas13d, hTERT, bladder cancer, aptazyme, degradation

## Abstract

Aptazyme and CRISPR/Cas gene editing system were widely used for regulating gene expression in various diseases, including cancer. This work aimed to reconstruct CRISPR/Cas13d tool for sensing hTERT exclusively based on the new device OFF-switch hTERT aptazyme that was inserted into the 3’ UTR of the Cas13d. In bladder cancer cells, hTERT ligand bound to aptamer in OFF-switch hTERT aptazyme to inhibit the degradation of Cas13d. Results showed that engineered CRISPR/Cas13d sensing hTERT suppressed cell proliferation, migration, invasion and induced cell apoptosis in bladder cancer 5637 and T24 cells without affecting normal HFF cells. In short, we constructed engineered CRISPR/Cas13d sensing hTERT selectively inhibited the progression of bladder cancer cells significantly. It may serve as a promising specifically effective therapy for bladder cancer cells.

## Introduction

Bladder cancer is one of the most common urologic neoplasms all over the world (Siegel et al., 2020). About 50% of bladder cancer patients will develop metastases within two years after diagnosis of bladder cancer (Sternberg et al., 2013). For bladder cancer patients with advanced metastasis, chemotherapy is the main treatment (Lenis et al., 2020). However, severe adverse reactions are caused due to poor specificity of chemotherapy drugs (Lenis et al., 2020). Thus, finding a highly specific targeted therapy for bladder cancer is of great value for bladder cancer patients.

Gene expression can be controlled by various tools including ligand-dependent small self-cleaving ribozymes (Lee et al., 2016). These ribozymes are named as aptazymes with properties of small, modular and no need of regulatory protein factors and have promising use in clinical applications (Felletti et al., 2016). Ribozyme platform, a communication module and aptamer are three main parts of the aptazymes (Nomura et al., 2012). The hammerhead ribozyme (HHR) is the common ribozyme platform (Zhong et al., 2016). When aptamer combines with ligand, the induced conformational change will be transferred to HHR via the communication module, generating cleavage activity (Spöring et al., 2020). OFF-switch and ON-switch are two types of aptazymes (Nomura et al., 2013; Beilstein et al., 2015; Yokobayashi, 2019). OFF-switch represents that gene expression is suppressed without corresponding ligand (Yokobayashi, 2019).

CRISPR/Cas13 is the class II type VI CRISPR (clustered regularly interspaced short palindromic repeats) gene editing tool (Huynh et al., 2020). A guide RNA (CRISPR-RNA, crRNA) and Cas13 are two components in this system (Huynh et al., 2020). It can target RNA substrate instead of DNA (Makarova et al., 2020). There are four subtypes of Cas13, including Cas13a, Cas13b, Cas13c and Cas13d and Cas13d is the smallest protein (Makarova et al., 2020). Compared with RNA interference (RNAi), CRISPR/Cas13 shows high efficiencies and on-target effects (O'Connell, 2019). CRISPR/Cas13 has been used in various fields. For example, Gootenberg et al. (2017) . had created a CRISPR/Cas13a-based molecular detection platform to distinguish genotype human DNA, pathogenic bacteria and identify mutations in cell-free tumor DNA (Gootenberg et al., 2017). Wang et al. (2019). reported that the formation of glioma intracranial tumors in mice was inhibited significantly using the collateral effect of CRISPR/Cas13a (Wang et al., 2019). A recent study showed that lung cancer cell viability was decreased significantly via CRISPR/Cas13a targeting EML4-ALK (Saifullah et al., 2020).

In our previous studies, we have shown that compared with normal cells, hTERT only existed in bladder cancer cells and it may be regarded as a specific ligand in bladder cancer (Liu et al., 2014; Huang et al., 2017). In this study, engineered CRISPR/Cas13d was constructed to selectively suppress the progression of bladder cancer via sensing hTERT ligand. The hTERT OFF-switch aptazyme was synthesized and inserted into the 3’UTR of Cas13d. MYC is an oncogene in bladder cancer and crRNA was designed to target MYC. As we expected, engineered CRISPR/Cas13d inhibited the mRNA and protein levels of MYC, and thus suppressed cell proliferation, migration, invasion and induced apoptosis of bladder cancer cells *in vitro*. However, there was no effects in normal human foreskin fibroblast (HFF) cells. In short, engineered CRISPR/Cas13d sensing hTERT may be another highly effective approach for kill bladder cancer cells specifically.

## Materials and Methods

### Cell Culture

Human foreskin fibroblast (HFF) was purchased from the Type Culture Collection of the Chinese Academy of Sciences, Shanghai, China. Human bladder cancer cell lines T24 and 5637 were purchased from the American Type Culture Collection (ATCC, Manassas, VA). T24 and 5637 were cultured from bladder cancer tissues with the histological grade of G3 and G2, respectively. These cells were cultured according to the manufacturer’s protocol.

### Cell Transfection

HFF or bladder cancer cells were seeded in plates. The corresponding plasmids were transfected into cells with Lipofectamine 3000 (Invitrogen, Carlsbad, CA, United States) according to the manufacturer’s instructions.

### RT-qPCR

TRIzol reagent was used to isolate total RNA from cells. A PrimeScript RT Reagent Kit with gDNA Eraser (Takara, Dalian, China) was utilized to synthesize the first strand of cDNA for detection of MYC and GAPDH. Quantitative PCR was then performed using the SYBR Premix Ex TaqTM kit (Takara, Dalian, China) on the Roche lightcycler 480 Real-Time PCR System. The comparative 2^−ΔΔCT^ method was used to analyze the expression levels.

### Vector Construction

Inactivated aptazyme sensing hTERT included hTERT aptamer and hammerhead ribozyme. The sequence of hTERT aptamer or hammerhead ribozyme was shown in the previous studies (Chen et al., 2010; Varshney et al., 2017). The sequence of hTERT aptamer is 5’-AGA​CAA​GAA​UAA​AAC​GCU​CAA​UAU​UGG​GCU​UUU​AGC​UUC​UUG​GUU​GGA​UAA​UAG​AUA​CAC​AUU​CGA​CAG​GAG​GCU​CAC​AAC​AGG​C-3’. The inactivated aptazyme sensing hTERT was inserted into 3’ UTR of the Cas13d (Addgene 109049) or downstream of the Renilla luciferase cassette in psiCHECK-2 (Promega). The crRNA targeting MYC mRNA was designed according to a previous study (Zhu et al., 2018). The sequence of crRNA used in this study was 5’-ACU​CGC​UGU​AGU​AAU​UCC​AGC​GAG​AGG​CA-3’.

### Luciferase Reporter Assay

The psiCHECK-2 vectors with inactivated aptazyme sensing hTERT were transfected into HFF or bladder cancer cells. Renilla and firefly luciferase activities were tested with the dual-luciferase reporter assay system (Promega, Madison, WI, United States) according to the user manuals.

### Cell Proliferation Assays

Cell Counting Kit-8 (CCK-8) assay and colony formation assay were used to detect engineered CRISPR/Cas13d on cell proliferation. For CCK-8 assay, 2,000 cells/well were cultured in 96-well plates. 10 μl CCK-8 reagent was added to each well for 0.5 h. The absorbance was measured at 450 nm using a microplate reader. For colony formation assay, 1,000 cells/well were plated onto six-well plates, and were incubated at 37°C and 5% CO_2_. After about 2°weeks, colonies were fixed using 0.05% crystal violet in 4% paraformaldehyde and counted using Image J program.

### Cell Apoptosis Assay

The FITC Annexin V Apoptosis Detection Kit (TransGen, Peking, China) was utilized to double stain cells with FITC-Annexin V and PI according to the manufacturer’s instructions. Right lower quadrant represents the percentage of early apoptosis cells.

### Cell Migration and Invasion Assays

100% confluence of bladder cancer/normal cells were scratched via a sterile 200 μl pipette tip. Images were taken from per well at 0 and 24 h. The migration distance between 0 and 24 h in each group was calculated. For cell invasion assay, cells were seeded to the upper chambers of the Transwell (Millicell, Merk KGaA). After 24 h, cells on the underside chambers were fixed in 4% paraformaldehyde for 30 min and stained with 0.1% crystal violet for 30 min and captured using a microscope. Quantification of the migrated cells was performed by counting cell numbers.

### Western Blot

RIPA Lysis Buffer (#P0013B; Beyotime) was used to extract protein. Protein concentrations were measured using Bicinchoninic Acid Kit (Sigma-Aldrich) according to the manufacturer’s protocol. Cell lysates were resolved by SDS-PAGE and transferred onto PVDF membranes. Membranes were blocked for 1 h with 5% non-fat milk in TBST and incubated overnight at 4°C with primary antibodies and required secondary antibodies conjugated to horseradish peroxidase and developed by chemiluminescent substrates.

### Statistical Analysis

All the above experiments were performed at least three times in this study. SPSS software version 22.0 (SPSS Inc., Chicago, IL, United States) was used to analyze all statistical analysis. All data are presented as the mean ± standard error (SD). Student’s *t*-test, was used to analyze the group difference. A *p*-value < 0.05 (two-sided) was regarded as statistical significance.

## Results

### The Expression of Renilla Luciferase Was Stable in Bladder Cancer Cells

**FIGURE 1:**
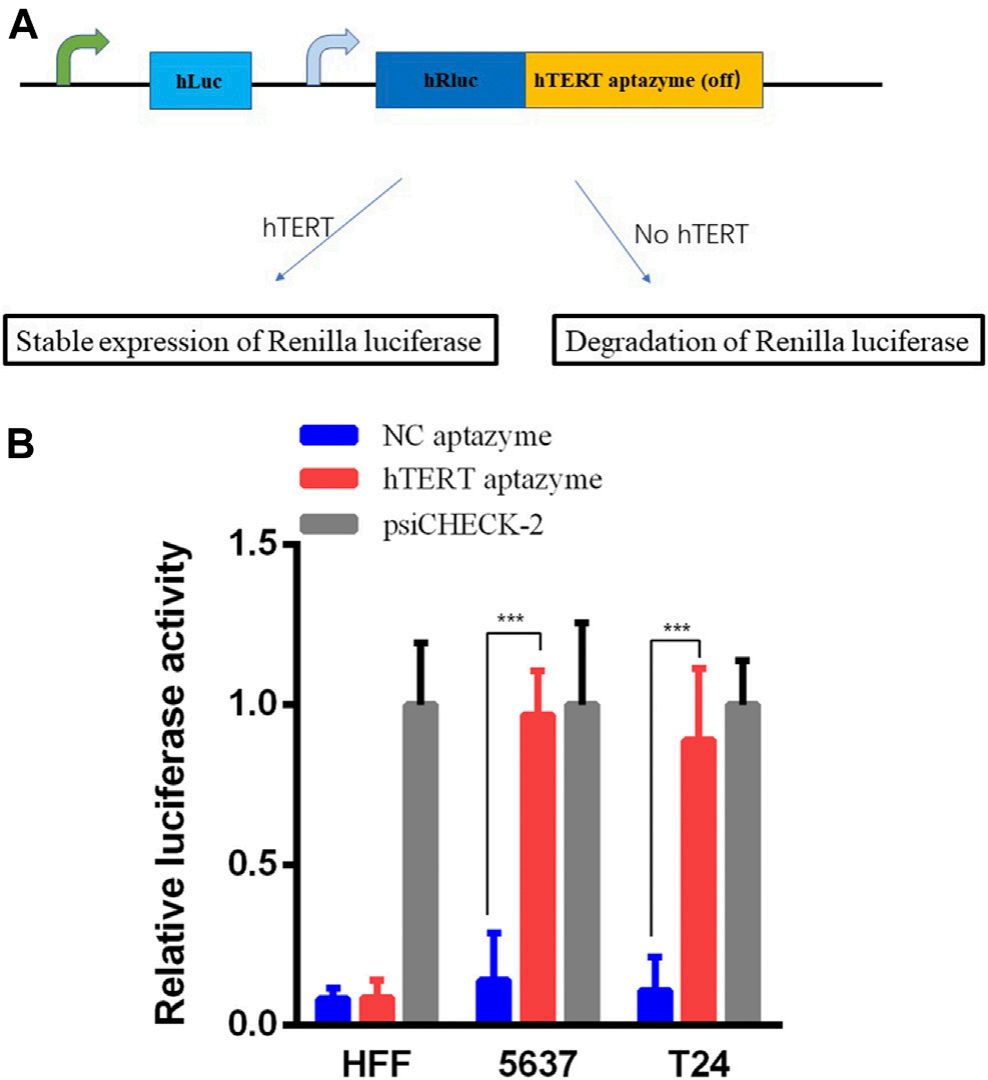
Relative luciferase activities were stable expressed in hTERT aptazyme group compared with NC aptazyme **(A)** The hTERT aptazyme was inserted into the 3’ UTR of Renilla luciferase gene and the schematic diagram of the hTERT aptazyme mode **(B)** Relative luciferase activities were stable expressed in hTERT aptazyme group in bladder cancer 5637 and T24 cells. However, Renilla luciferase was degraded significantly in normal cell HFF. *** represents *p* < 0.001.

The hTERT OFF-switch aptazyme was constructed as described in the Materials and Methods section. The hTERT aptazyme was synthesized and inserted into the 3’UTR of Renilla luciferase gene in psiCHECK-2 vector (Figure 1A). As shown in Figure 1B, compared with the NC aptazyme group, the relative luciferase activities of hTERT aptazyme group were no difference in HFF. However, the relative luciferase activities of hTERT aptazyme group was increased significantly in bladder cancer 5637 and T24 cells. It represents that hTERT ligand bound to OFF-switch aptazyme and restrained the degradation of Renilla luciferase.

### Engineered CRISPR/Cas13d Sensing hTERT Selectively Suppressed the mRNA and Protein Levels of MYC

**FIGURE 2:**
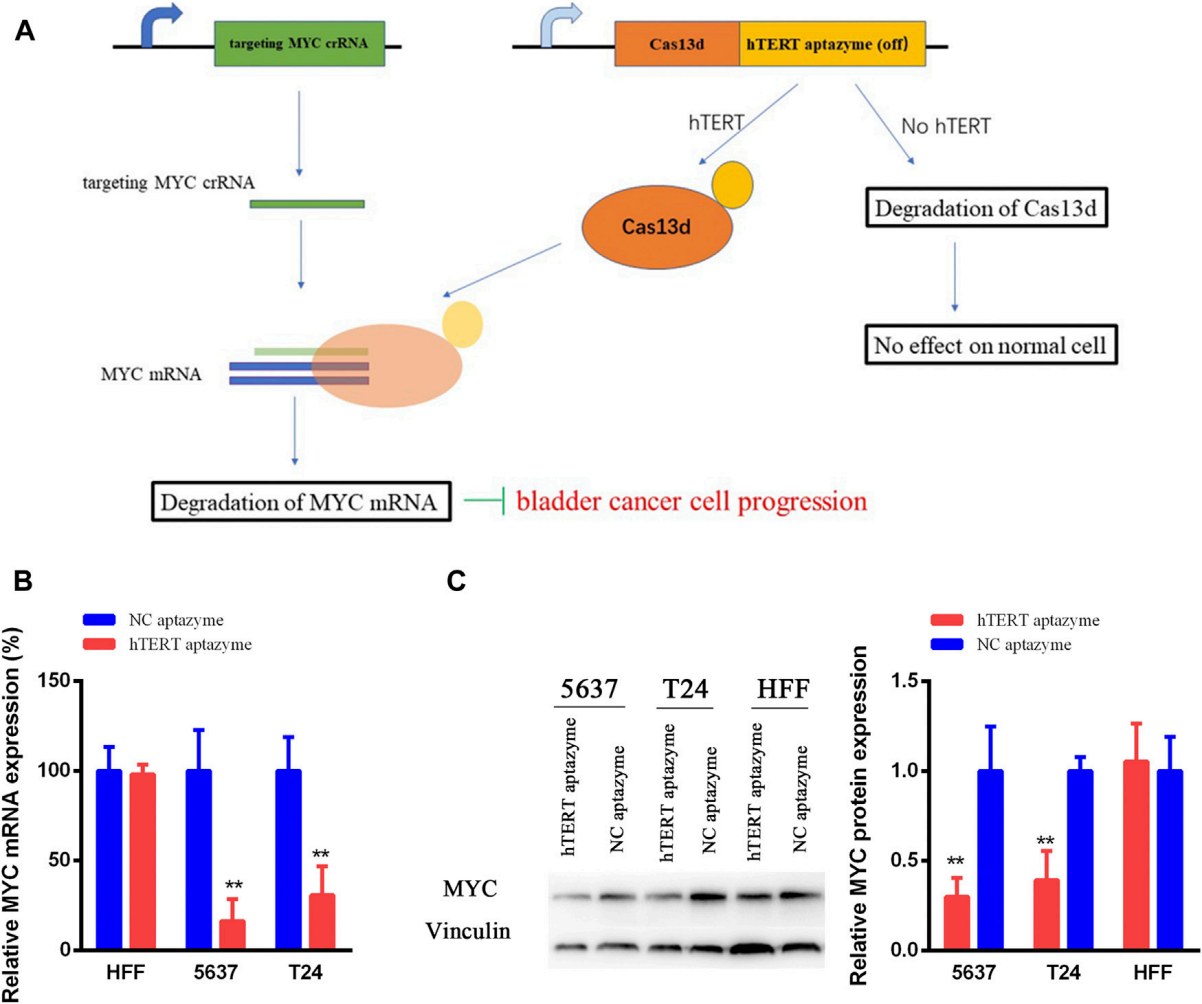
Schematic diagram of engineered CRISPR/Cas13d sensing hTERT in bladder cancer **(A)** The working mechanism of engineered CRISPR/Cas13d sensing hTERT in bladder cancer cells **(B and C)** Engineered CRISPR/Cas13d sensing hTERT selectively suppressed the MYC mRNA and protein expression levels without affecting HFF. *represents *p* < 0.05, ** means *p* < 0.01.

As shown in schematic diagram in Figure 2A, the sequence of hTERT OFF-switch aptazyme was inserted into the 3’UTR of Cas13d. The crRNA targeting oncogene MYC mRNA was designed. In HFF, the expression of hTERT ligand was very low or loss, and induces the degradation of Cas13d. However, Cas13d is highly expressed in cancer cells and with the guidance of crRNA targeting MYC mRNA, Cas13d binds to MYC mRNA results in degradation of MYC expression at mRNA and protein levels. Finally, the progression of bladder cancer is suppressed. In order to validate this mechanism of engineered CRISPR/Cas13d sensing hTERT, we detected the mRNA and protein expression levels of MYC. The MYC mRNA expression levels were decreased significantly between NC aptazyme and hTERT aptazyme group in bladder cancer 5637 and T24 cells. However, it was no difference in HFF (Figure 2B). Similarly, the MYC protein levels were selectively inhibited markedly in bladder cancer 5637 and T24 cells except HFF. In short, the mRNA and protein levels of MYC were restrained selectively in bladder cancer without affecting normal cells via engineered CRISPR/Cas13d sensing hTERT.

### Bladder Cancer Cell Proliferation Was Selectively Inhibited by Engineered CRISPR/Cas13d Sensing hTERT

**FIGURE 3:**
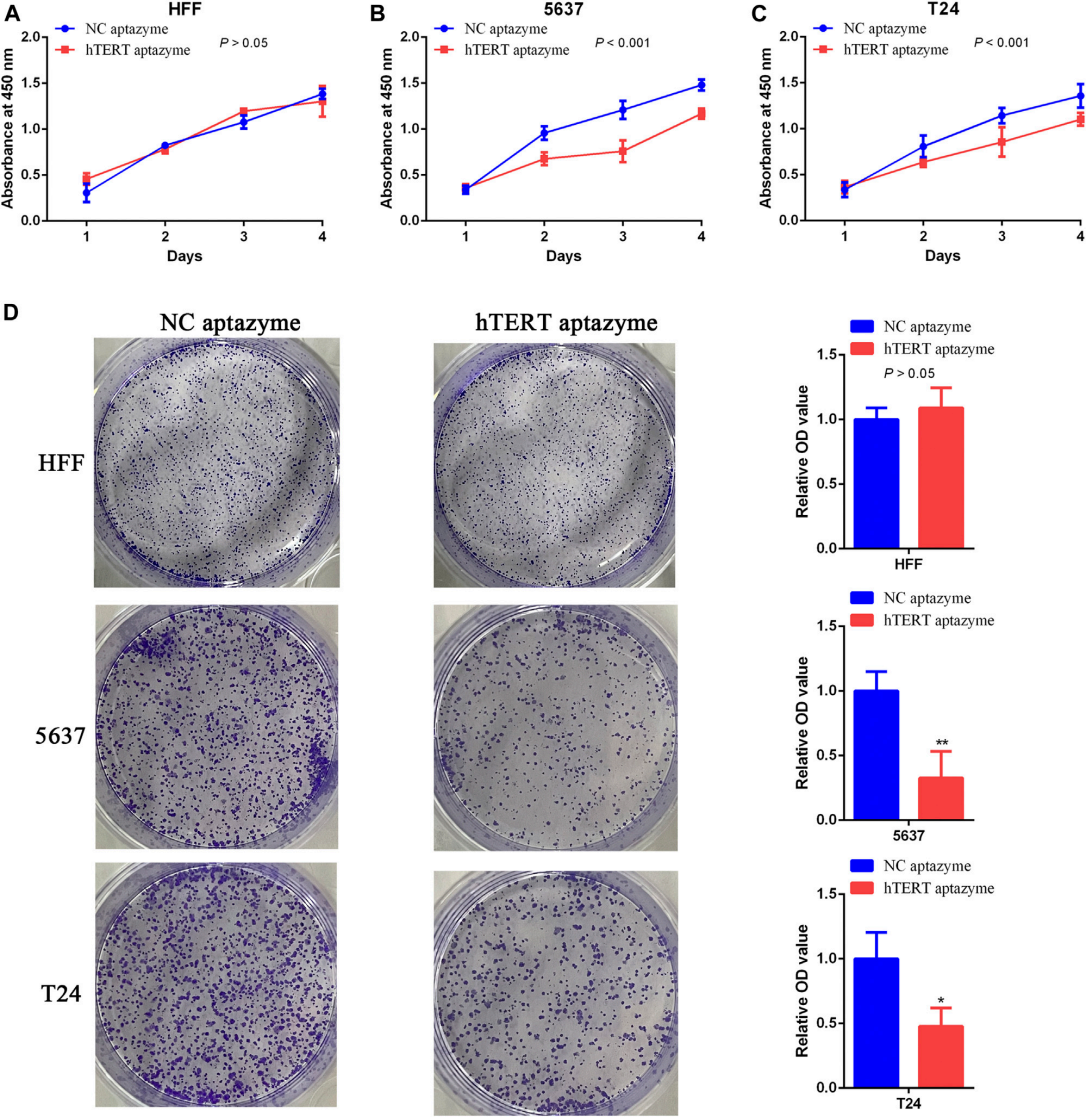
Engineered CRISPR/Cas13d sensing hTERT selectively inhibited bladder cancer cell proliferation **(A–C)** Cell growth was suppressed by engineered CRISPR/Cas13d sensing hTERT without affecting HFF through CCK-8 assay **(D)** Results of colony formation assay illustrated that bladder cancer cell proliferation was selectively inhibited via engineered CRISPR/Cas13d sensing hTERT.

Next, the effects of engineered CRISPR/Cas13d sensing hTERT were detected in bladder cancer cells using CCK-8 and colony formation assay. Cell growth was not changed in HFF between NC aptazyme and hTERT aptazyme group (Figure 3A). However, compared with NC aptazyme set, cell proliferation of bladder cancer 5637 and T24 cells was suppressed significantly (Figures 3B,C). Analogously, colony formation was no difference between these two objects. Nonetheless, bladder cancer 5637 and T24 cell colony was inhibited dramatically between NC aptazyme and hTERT aptazyme group (Figure 3D). All in all, these results demonstrated that engineered CRISPR/Cas13d sensing hTERT selectively restrained bladder cancer cell proliferation.

### Bladder Cancer Cell Apoptosis Was Selectively Promoted by Engineered CRISPR/Cas13d Sensing hTERT

**FIGURE 4:**
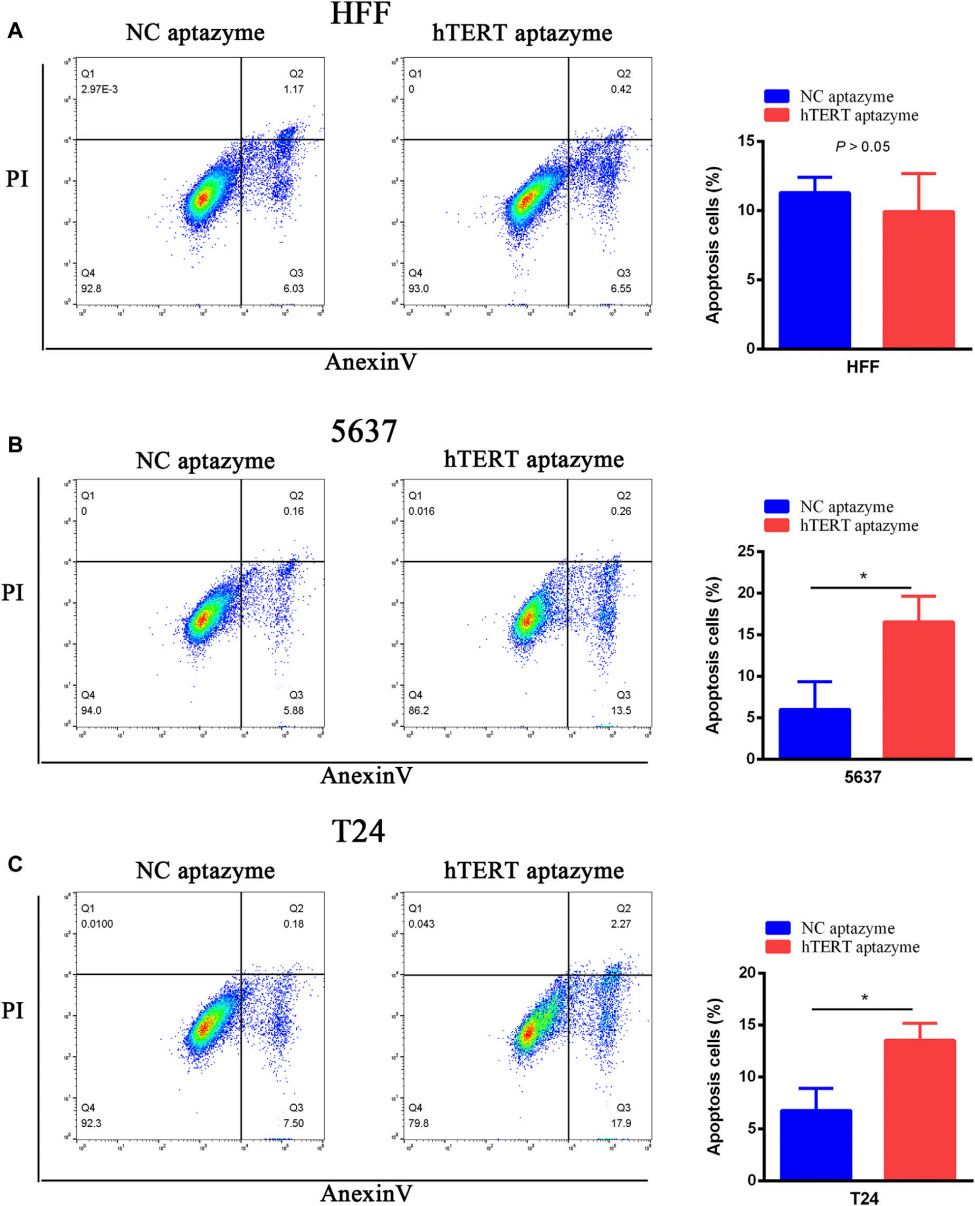
Engineered CRISPR/Cas13d sensing hTERT selectively induced bladder cancer cell apoptosis. Engineered CRISPR/Cas13d sensing hTERT have no effects on apoptosis in HFF **(A)**. However, cell apoptosis was significantly increased by engineered CRISPR/Cas13d sensing hTERT in bladder cancer 5637 **(B)** and T24 **(C)** cells.

The FITC Annexin V Apoptosis Detection Kit was used to measure the effects of engineered CRISPR/Cas13d on cell apoptosis in bladder cancer cells. As shown in Figure 4A, cell apoptosis was not changed between NC aptazyme and hTERT aptazyme group. However, the cell apoptosis of hTERT aptazyme group was increased significantly compared with NC aptazyme object in bladder cancer 5637 cells (Figure 4B). Homoplastically, engineered CRISPR/Cas13d sensing hTERT promoted apoptosis obviously in bladder cancer T24 cells compared with the negative control (Figure 4C). In short, the above results illustrated that engineered CRISPR/Cas13d sensing hTERT selectively promoted bladder cancer cell apoptosis.

### Bladder Cancer Cell Migration and Invasion Were Selectively Suppressed by Engineered CRISPR/Cas13d Sensing hTERT

**FIGURE 5:**
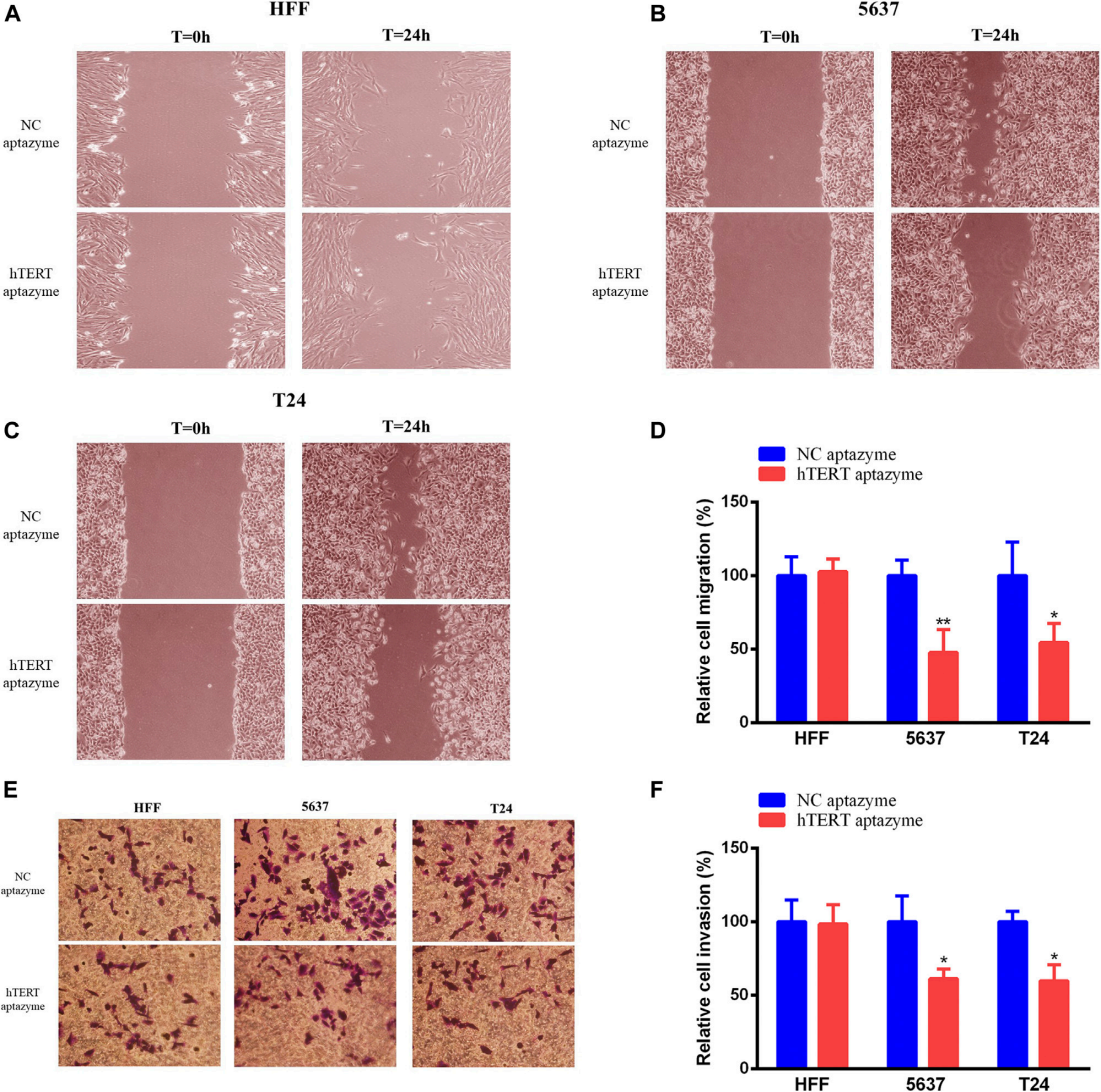
Engineered CRISPR/Cas13d sensing hTERT selectively suppressed bladder cancer cell migration and invasion. Engineered CRISPR/Cas13d sensing hTERT have no effects on cell migration and invasion in HFF **(A,E,F)**. However, cell migration and invasion were significantly suppressed by engineered CRISPR/Cas13d sensing hTERT in bladder cancer 5637 and T24 cells **(B–F)**.

Engineered CRISPR/Cas13d sensing hTERT had no effects on cell migration and invasion in HFF and bladder cancer cells (Figures 5A,D–F). However, cell migration and invasion were inhibited significantly via engineered CRISPR/Cas13d in bladder cancer 5637 and T24 cells (Figures 5B–F). These results demonstrated that engineered CRISPR/Cas13d sensing hTERT could selectively inhibit bladder cancer cell migration and invasion.

## Discussion

The role of hTERT was in-depth study in recent years and hTERT involves in the development of diseases including cancer. The hTERT is highly expressed in all human cancers except normal human cells (not include embryonic stem cells and germ cells) (Chen et al., 2020). Studies have demonstrated that hTERT maintains cancer cell immortalization and involves closely in cancer growth, metastasis and transformation (Liu et al., 2017; Chen et al., 2018b). Lots of studies have reported that hTERT is a promising cancer biomarker in various kinds of cancer (Shi et al., 2014; Chen et al., 2017). In our previous studies (Huang et al., 2017; Zhuang et al., 2017), we have validated that hTERT was only expressed in bladder cancer cells except normal cells HFF. The strategy of utilization of hTERT will be a valuable method to distinguish bladder cancer cells and normal cells.

Artificial riboswitch (aptazyme) has been used to regulate gene expression precisely via binding between RNA and ligand (Pu et al., 2020). Aptazyme was utilized to control mRNA cleavage through self-cleavage within the mRNA (Takahashi and Yokobayashi, 2019; Spöring et al., 2020). Aptazyme can be inserted into 5’ or 3’ UTR of gene mRNA for controlling gene expression (Chen et al., 2018a). A previous study has reported that an optimal hTERT aptamer was synthesized, screened and can exclusively bind to hTERT *in vitro* and *in vivo* (Varshney et al., 2017).

Various studies demonstrated that CRISPR/Cas gene editing tools have been used for gene expression in cancer (Sharma et al., 2020). Targeting DNA of CRISPR/Cas9 system was widely reconstructed to create new gene circuits for cancer treatment (Liu et al., 2020). However, high off-target effect is inevitable for CRISPR/Cas9 (Pruett-Miller, 2020). CRISPR/Cas13d was another gene editing method to targeting RNA molecules (Lin et al., 2020). It showed higher efficiencies and on-target effects in cells (Lin et al., 2020). In this study, we synthesized the OFF-switch hTERT aptazyme and inserted into 3’UTR of the Cas13d according to previous studies. When hTERT existed in cells, hTERT bound to OFF-switch hTERT aptazyme and restrained the degradation of Cas13d. On the contrary, Cas13d was degraded without hTERT in normal cells. This engineered CRISPR/Cas13d sensing hTERT was tested in bladder cancer 5637 and T24 cells in this subject. Results showed that engineered CRISPR/Cas13d sensing hTERT selectively suppressed the progression of bladder cancer cells except normal cell HFF. However, deficiencies of this study still existed. The protein expression levels of Cas13d were not shown in this study owing to lack of Cas13d antibody at present. The role of engineered CRISPR/Cas13d sensing hTERT *in vivo* is lack. We will further confirm this *in vivo* effect in the near future.

In short, engineered CRISPR/Cas13d sensing hTERT was constructed and selectively suppressed the progression of bladder cancer cells. It may provide a promising precise exclusively method for bladder cancer.

### Data Availability Statement

The original contributions presented in the study are included in the article/Supplementary Material, further inquiries can be directed to the corresponding authors.

## References

[B1] Beilstein K. Wittmann A. Grez M. Suess B. (2015). Conditional control of mammalian gene expression by tetracycline-dependent hammerhead ribozymes. ACS Synth. Biol. 4, 526–534. 10.1021/sb500270h 25265236

[B2] Chen Y. Y. Jensen M. C. Smolke C. D. (2010). Genetic control of mammalian T-cell proliferation with synthetic RNA regulatory systems. Proc. Natl. Acad. Sci. USA 107, 8531–8536. 10.1073/pnas.1001721107 20421500 PMC2889348

[B3] Chen P. Gu W.-L. Gong M.-Z. Wang J. Li D.-Q. (2017). shRNA-mediated silencing of hTERT suppresses proliferation and promotes apoptosis in osteosarcoma cells. Cancer Gene Ther. 24, 325–332. 10.1038/cgt.2017.22 28799566

[B4] Chen H. Li Y. Du C. Li Y. Zhao J. Zheng X. (2018a). Aptazyme-mediated direct modulation of post-transcriptional sgRNA level for conditional genome editing and gene expression. J. Biotechnol. 288, 23–29. 10.1016/j.jbiotec.2018.10.011 30391232

[B5] Chen L. Chen C. Chen W. Li K. Chen X. Tang X. (2018b). Biodegradable black phosphorus nanosheets mediate specific delivery of hTERT siRNA for synergistic cancer therapy. ACS Appl. Mater. Inter. 10, 21137–21148. 10.1021/acsami.8b04807 29882656

[B6] Chen K. Li L. Qu S. Pan X. Sun Y. Wan F. (2020). Silencing hTERT attenuates cancer stem cell-like characteristics and radioresistance in the radioresistant nasopharyngeal carcinoma cell line CNE-2R. Aging 12, 25599–25613. 10.18632/aging.104167 33234740 PMC7803545

[B7] Felletti M. Stifel J. Wurmthaler L. A. Geiger S. Hartig J. S. (2016). Twister ribozymes as highly versatile expression platforms for artificial riboswitches. Nat. Commun. 7, 12834. 10.1038/ncomms12834 27670347 PMC5052635

[B8] Gootenberg J. S. Abudayyeh O. O. Lee J. W. Essletzbichler P. Dy A. J. Joung J. (2017). Nucleic acid detection with CRISPR-Cas13a/C2c2. Science 356, 438–442. 10.1126/science.aam9321 28408723 PMC5526198

[B9] Huang X. Zhuang C. Zhuang C. Xiong T. Li Y. Gui Y. (2017). An enhanced hTERT promoter-driven CRISPR/Cas9 system selectively inhibits the progression of bladder cancer cells. Mol. Biosyst. 13, 1713–1721. 10.1039/c7mb00354d 28702647

[B10] Huynh N. Depner N. Larson R. King-Jones K. (2020). A versatile toolkit for CRISPR-Cas13-based RNA manipulation in drosophila. Genome Biol. 21, 279. 10.1186/s13059-020-02193-y 33203452 PMC7670108

[B11] Lee C. H. Han S. R. Lee S.-W. (2016). Therapeutic applications of aptamer-based riboswitches. Nucleic acid Ther. 26, 44–51. 10.1089/nat.2015.0570 26539634

[B12] Lenis A. T. Lec P. M. Chamie K. Mshs M. D. (2020). Bladder cancer: a review. Jama 324, 1980–1991. 10.1001/jama.2020.17598 33201207

[B13] Lin P. Qin S. Pu Q. Wang Z. Wu Q. Gao P. (2020). CRISPR-Cas13 inhibitors block RNA editing in bacteria and mammalian cells. Mol. Cel. 78, 850–861. 10.1016/j.molcel.2020.03.033 PMC7299153 32348779

[B14] Liu Y. Zeng Y. Liu L. Zhuang C. Fu X. Huang W. (2014). Synthesizing AND gate genetic circuits based on CRISPR-Cas9 for identification of bladder cancer cells. Nat. Commun. 5, 5393. 10.1038/ncomms6393 25373919

[B15] Liu T. Li W. Lu W. Chen M. Luo M. Zhang C. (2017). RBFOX3 promotes tumor growth and progression via hTERT signaling and predicts a poor prognosis in hepatocellular carcinoma. Theranostics 7, 3138–3154. 10.7150/thno.19506 28839469 PMC5566111

[B16] Liu Y. Huang W. Cai Z. (2020). Synthesizing AND gate minigene circuits based on CRISPReader for identification of bladder cancer cells. Nat. Commun. 11, 5486. 10.1038/s41467-020-19314-7 33127914 PMC7599332

[B17] Makarova K. S. Wolf Y. I. Iranzo J. Shmakov S. A. Alkhnbashi O. S. Brouns S. J. J. (2020). Evolutionary classification of CRISPR-Cas systems: a burst of class 2 and derived variants. Nat. Rev. Microbiol. 18, 67–83. 10.1038/s41579-019-0299-x 31857715 PMC8905525

[B18] Nomura Y. Kumar D. Yokobayashi Y. (2012). Synthetic mammalian riboswitches based on guanine aptazyme. Chem. Commun. (camb). 48, 7215–7217. 10.1039/c2cc33140c 22692003

[B19] Nomura Y. Zhou L. Miu A. Yokobayashi Y. (2013). Controlling mammalian gene expression by allosteric hepatitis delta virus ribozymes. ACS Synth. Biol. 2, 684–689. 10.1021/sb400037a 23697539 PMC3874218

[B20] O'Connell M. R. (2019). Molecular mechanisms of RNA targeting by cas13-containing type VI CRISPR-cas systems. J. Mol. Biol. 431, 66–87. 10.1016/j.jmb.2018.06.029 29940185

[B21] Pruett-Miller S. M. (2020). Assessing off-target editing of CRISPR-cas9 systems. CRISPR J. 3, 430–432. 10.1089/crispr.2020.29116.smi 33346715

[B22] Pu Q. Zhou S. Huang X. Yuan Y. Du F. Dong J. (2020). Intracellular selection of theophylline-sensitive hammerhead aptazyme. Mol. Ther. Nucleic Acids 20, 400–408. 10.1016/j.omtn.2020.03.001 32244167 PMC7118274

[B23] Saifullah Sakari. M. Suzuki T. Yano S. Tsukahara T. (2020). Effective RNA knockdown using CRISPR-cas13a and molecular targeting of the EML4-ALK transcript in H3122 lung cancer cells. Int. J. Mol. Sci. 21, 8904. 10.3390/ijms21238904 PMC7727695 33255340

[B24] Sharma G. Sharma A. R. Bhattacharya M. Lee S. S. Chakraborty C. (2020). CRISPR-Cas9: a preclinical and clinical perspective for the treatment of human diseases. Mol. Ther. 29, 571–586. 10.1016/j.ymthe.2020.09.028 33238136 PMC7854284

[B25] Shi Y.-A. Zhao Q. Zhang L.-H. Du W. Wang X.-Y. He X. (2014). Knockdown of hTERT by siRNA inhibits cervical cancer cell growth *in vitro* and *in vivo* . Int. J. Oncol. 45, 1216–1224. 10.3892/ijo.2014.2493 24920549

[B26] Siegel R. L. Miller K. D. Jemal A. (2020). Cancer statistics, 2020. CA: Cancer J. Clin. 70, 7–30. 10.3322/caac.21590 31912902

[B27] Spöring M. Boneberg R. Hartig J. S. (2020). Aptamer-Mediated control of polyadenylation for gene expression regulation in mammalian cells. ACS Synth. Biol. 9, 3008–3018. 10.1021/acssynbio.0c00222 33108164

[B28] Sternberg C. N. Bellmunt J. Sonpavde G. Siefker-Radtke A. O. Stadler W. M. Bajorin D. F. (2013). ICUD-EAU international consultation on bladder cancer 2012: chemotherapy for urothelial carcinoma-neoadjuvant and adjuvant settings. Eur. Urol. 63, 58–66. 10.1016/j.eururo.2012.08.010 22917984

[B29] Takahashi K. Yokobayashi Y. (2019). Reversible gene regulation in mammalian cells using riboswitch-engineered vesicular stomatitis virus vector. ACS Synth. Biol. 8, 1976–1982. 10.1021/acssynbio.9b00177 31415142

[B30] Varshney A. Bala J. Santosh B. Bhaskar A. Kumar S. Yadava P. K. (2017). Identification of an RNA aptamer binding hTERT-derived peptide and inhibiting telomerase activity in MCF7 cells. Mol. Cel Biochem 427, 157–167. 10.1007/s11010-016-2907-7 28004350

[B31] Wang Q. Liu X. Zhou J. Yang C. Wang G. Tan Y. (2019). The CRISPR‐cas13a gene‐editing system induces collateral cleavage of RNA in glioma cells. Adv. Sci. (Weinh) 6, 1901299. 10.1002/advs.201901299 31637166 PMC6794629

[B32] Yokobayashi Y. (2019). Aptamer-based and aptazyme-based riboswitches in mammalian cells. Curr. Opin. Chem. Biol. 52, 72–78. 10.1016/j.cbpa.2019.05.018 31238268 PMC7108311

[B33] Zhong G. Wang H. Bailey C. C. Gao G. Farzan M. (2016). Rational design of aptazyme riboswitches for efficient control of gene expression in mammalian cells. eLife 5, 18858. 10.7554/elife.18858 PMC5130294 27805569

[B34] Zhuang C. Huang X. Zhuang C. Luo X. Zhang X. Cai Z. (2017). Synthetic regulatory RNAs selectively suppress the progression of bladder cancer. J. Exp. Clin. Cancer Res. CR 36, 151. 10.1186/s13046-017-0626-x 29084575 PMC5663129

[B35] Zhu H. Richmond E. Liang C. (2018). CRISPR-RT: a web application for designing CRISPR-C2c2 crRNA with improved target specificity. Bioinformatics 34, 117–119. 10.1093/bioinformatics/btx580 28968770

